# The perspectives of speech–language pathologists: Providing teletherapy to patients with speech, language and swallowing difficulties during a COVID-19 context

**DOI:** 10.4102/sajcd.v69i2.902

**Published:** 2022-08-11

**Authors:** Zahraa Tar-Mahomed, Kelly-Ann Kater

**Affiliations:** 1 Department of Speech Pathology and Audiology, Faculty of Humanities, School of Human and Community Development, University of the Witwatersrand, Johannesburg, South Africa

**Keywords:** teletherapy, stroke, communication disorders, swallowing disorders, speech therapy, speech–language pathologist (SLP), stroke intervention

## Abstract

**Background:**

The coronavirus disease 2019 (COVID-19) pandemic has had a huge impact on every facet of life. This directly included the delivery of health care from allied health professionals such as speech–language pathologists (SLPs) in South Africa. Research has shown that there is limited research done locally on the impact of COVID-19 relating to stroke care. Consequently, this results in a lack of research on the provision of speech, language and swallowing intervention using teletherapy after a stroke from an SLP point of view.

**Objectives:**

The aim of this study was to explore the experiences of SLPs with regard to their use of teletherapy in a COVID-19 context when providing speech, language and swallowing intervention for patients after a stroke.

**Methods:**

This study made use of a qualitative approach. An electronic questionnaire was sent to SLPs inviting them to participate in the study. Purposive sampling was used to recruit participants and thematic content analysis was used to analyse the open-ended qualitative questions.

**Results:**

The findings show that SLPs experienced a variety of facilitators and barriers to using teletherapy. Additionally, issues of access differ across the private and public sector SLPs for both the clients and the SLPs.

**Conclusion:**

The current study provided research in the field of teletherapy, which is relatively new in the South African context. The study, whilst small in scale, provided some insight into the changes experienced from the shift to teletherapy.

## Introduction

Speech–language pathologists (SLPs) play an integral role in the screening, assessment, management and rehabilitation of stroke survivors who present with dysphagia, communication and/or cognitive-linguistic impairments. Early diagnosis and referral to an SLP is critical, as is intensive intervention as soon as the patient is able to participate. This helps to ensure better patient outcomes (Dilworth, 2008). With the current coronavirus disease 2019 (COVID-19) pandemic and the shift to teletherapy being relatively new in the South African context, it is important to understand the various barriers and facilitators to health care that may affect the service delivery of SLPs.

South Africa had its first COVID-19 case on 06 March 2020, and on 27 March 2020, the government instituted a nationwide level 5 lockdown. Restrictions on movement during the level 5 lockdown specifically required individuals to remain in their place of residence, with the exceptions of those performing an essential service, obtaining essential goods or services, collecting a social grant or pension or seeking emergency, life-saving or chronic medical attention (Siedner et al., 2020). As hospitals struggled with an increase in caseloads, many health care professionals took on additional roles and outpatient services were either suspended or delivered via teletherapy (Adams, Seedat, Coutts, & Kater, 2021).

Based on the research done on telerehabilitation, it was found that it has the ability to change the delivery of health care beyond stroke to other neurological conditions (Tenforde et al., 2020). This may be different in the South African context, where factors such as socio-economic status, culture, education and so on need to be considered. Research shows that there are various barriers to the implementation of telerehabilitation in the public sector and telerehabilitation in general in South Africa (Mars, 2011). This is because telerehabilitation requires information and communication technologies, their associated infrastructure and electricity. In the context of South Africa, technology problems such as unreliable electricity supply and low bandwidth are barriers to the successful implementation of telemedicine (Cilliers & Flowerday, 2014). Telemedicine in Africa is approximately 10 years behind the developed world (Mars, 2011).

Research suggests that the service delivery of allied health care to rural or remote communities displays a huge problem in many countries (Lincoln, Hines, Fairweather, Ramsden, & Martinovich, 2014). In a developing country like South Africa, this is even more prevalent (Maphumulo & Bhengu, 2019). Telerehabilitation may overcome patient anxieties and is seen as being more cost effective and safe (Eron, 2010). Telerehabilitation avoids the use of personal protective equipment (PPE), reduces the risk for the stroke team, allows a reasonable stroke evaluation and avoids unnecessary interfacility transfers (Markus & Brainin, 2020).

The role of the SLP in providing therapy for stroke patients is well known in terms of face-to-face intervention; however, the lines become blurred when it comes to managing the consequences of stroke using teletherapy in a context like South Africa during COVID-19. There is research that has been done on stroke care using telerehabilitation that is not specific to the COVID-19 and the South African context but which can be applied. A study done by Tchero, Tabue-Teguo, Lannuzel and Rusch (2018) illustrates that telerehabilitation can be a suitable alternative to usual rehabilitation care in poststroke patients, especially in remote or underserved areas.

At the beginning of the COVID-19 pandemic, the South African Government decreed all health workers (which include rehabilitation services) part of essential services (South African Speech–Language and Hearing Association [SASLHA], 2020). The South African Speech–Language and Hearing Association consequently stated that the profession of speech–language pathology is an essential service with emphasis on the fact that communicating and eating are basic human rights. This has led to an increase in terms of the use of teletherapy in South Africa since COVID-19 and was found to have huge benefits to many patients with communication, hearing and swallowing disabilities (Adams et al., 2021; SASLHA, 2020).

The aim of stroke rehabilitation is to reduce the disabilities and enable the patient to return to the community. However, with the implementation of the lockdown restrictions and the subsequent cessation of face-to-face therapeutic interventions, South African SLPs had to find ways to maintain essential services.

The impairments associated with a stroke exhibit a wide diversity of clinical signs and symptoms. Disability, which is multifactorial in its determination, varies according to the degree of neurological recovery, the site of the lesion, the patient’s premorbid status and the environmental support systems (Teasell & Hussein, 2016). In addition to the physical impairments caused by stroke, many individuals may experience changes in their cognitive, communication and swallowing abilities (Dragga, 2015). Dysphagia and communication impairment are common consequences of stroke. Stroke survivors with either or both of these impairments are likely to have poorer long-term outcomes than those who do not have them (Dilworth, 2008).

Speech–language pathologists are trained to evaluate and treat disorders such as communication disorders, swallowing difficulties (dysphagia) and cognitive disorders that are a consequence of stroke. They are an integral part of the rehabilitation team (Dragga, 2015). They also play a role in counselling both patients and their family members. Additionally, they play an important role as part of a multidisciplinary team and may need to consult with other professionals to improve patient outcomes (Desrosiers et al., 2002). The communication deficits associated with stroke are diverse; they can be described as affecting one or more of these areas: language, motor speech, swallowing and/or cognitive communication. An assessment is conducted to identify how these areas are affected post stroke. This is achieved by gaining a full picture of the patients’ life before the injury through a case history. A thorough assessment is then conducted and a therapy programme is devised for the patient (Dilworth, 2008). Communication disorders in adults can have a significant effect on their quality of life and that of their families. Speech–language pathologists face several challenges in providing assessment and treatment services to such people. Challenges include facilitating equitable access to services and providing appropriate management within a changing social and economic context (Theodoros, 2008).

The aim of stroke rehabilitation is to reduce the disabilities and enable the patient to return to the community. This is achieved by joint efforts of physicians, physiotherapists, occupational therapists, SLPs, nurses, social workers and psychologists (Nair & Taly, 2002). Rehabilitation of a person who had a stroke begins as soon as any impairment is perceived and comprises traditional exercise programmes as well as neuropsychological approaches with the primary aim of restoring patient mobility. It also deals with issues related to dysphagia, which is where the SLP comes in (Nair & Taly, 2002).

Understanding the impact of COVID-19 on SLPs has a three-fold purpose: to re-evaluate service provision, to reassess service delivery platforms and to identify the need for support to SLPs during a time of crisis. Given the uncertainty around the development, acceptance and roll-out of a vaccine for COVID-19, the country as a whole needed to ensure that the impact on future health service provision was minimised. Therefore, South Africa needed to find ways to create surge capacity to treat COVID-19 patients whilst maintaining essential services (Adams et al., 2021). It is well known that South Africa’s health care provision in private and public is rife with inequalities, but in some ways, COVID-19 may have levelled the playing fields regarding resource allocation of health care services because of the above (Labuschaigne, 2020). Despite the increase in use of teletherapy in South Africa since COVID-19, there are certain disadvantages and advantages that accompany this new practice (Mars, 2011).

This study aimed to look at the shift from face-to-face therapy to teletherapy and the various concerns that come with it for SLPs treating speech, language or swallowing disorders after a stroke. It was important to conduct this research in the South African context as it allowed one to explore the barriers and facilitators to teletherapy from the perspective of the SLPs, especially as South Africa is such a diverse nation and there are other factors that come into play, such as cultural and language differences that may impact the experiences of the SLPs in using teletherapy (Adams et al., 2021).

## Method

The aims of the study were to describe the facilitators and barriers of providing speech, language and swallowing intervention via teletherapy, as well as to describe the access of South African SLPs in providing teletherapy for a person who had a stroke. A qualitative design was used.

**FIGURE 1:**
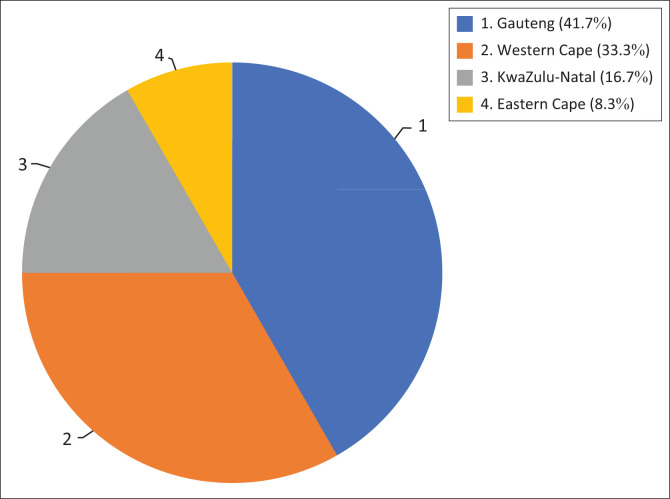
Number of participants per province.

**FIGURE 2:**
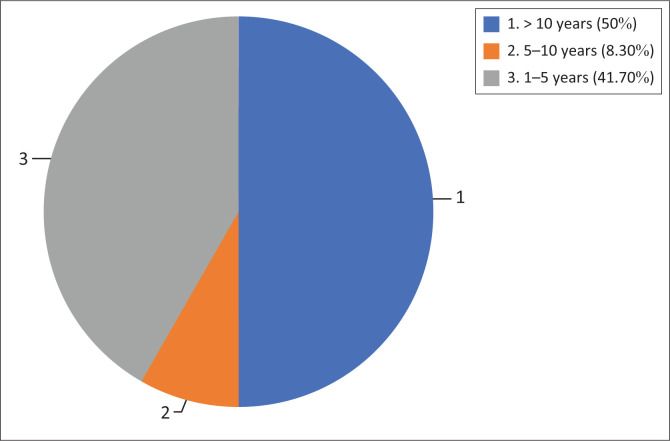
Participants’ years of experience.

The researcher aimed to have a sample size of 20–30 participants (Peytchev, 2013). Selecting a larger sample size would have provided a better representation of the population targeted in the study (Peytchev, 2013). However, it proved difficult to obtain an adequate number of responses, as it could not be guaranteed that every individual invited to participate in the study would respond. Because of the current circumstances of the COVID-19 pandemic, where aspects such as survey fatigue become prominent, only 12 participants were obtained. The demographics of the participants are illustrated in Figure 1 and Figure 2.

Despite the questionnaire being circulated to SLPs in all provinces, only SLPs from Gauteng, the Western Cape, KwaZulu-Natal and the Eastern Cape participated in this study. This could be because the Western Cape, Gauteng, KwaZulu-Natal and the Eastern Cape have considerably higher practitioner per 10 000 population ratios than the North West and Northern Cape (Pillay, Tiwari, Kathard, & Chikte, 2020).

Furthermore, 75% of these participants practise in the private sector, whereas only 25% practise in the public sector. This links to previous research where it was found that nationally, only 22% of SLPs are employed in the public sector (Pillay et al., 2020).

Data collection occurred by means of a self-administered questionnaire (Appendix 1) which was circulated via Google forms. The questions on the self-administered questionnaire aligned with the overall research question, as well as the objectives of the study. The questionnaire was developed by the researcher and was made up of 22 questions, which were a combination of closed-ended and open-ended questions. A pilot study was conducted whereby the length of the questionnaire and appropriateness of the questions in relation to the aims were determined. The questionnaire began with demographic questions and later probed the advantages and disadvantages in terms of the provision of SLP services with regard to stroke intervention using teletherapy. Once data were collected and responses were transcribed, the data were then analysed using thematic content analysis (Clarke, Braun, & Hayfield, 2015).

### Ethical considerations

Following ethical approval from the Institutional Review at a large Gauteng public university (reference number: STA_2020_32), participants were recruited via purposive sampling. All participants had to be registered with the Health Professionals Council of South Africa (HPCSA) and working with stroke patients. Participants were contacted and invited to participate in the study through electronic mail and links on social media as well as via SASHLA in order to reach as many SLPs as possible. Speech–language pathologists from both the public and private sector were invited to participate in the study.

## Results and discussion

The aim of this study was to provide a description of the experience of SLPs who provided teletherapy to stroke survivors in a COVID-19 context. Findings from this study suggest that SLPs perceived the delivery of teletherapy to be feasible and worthwhile, despite facing some challenges with technology and connectivity.

## Facilitators and advantages of providing speech, language and swallowing intervention via teletherapy

Participants mentioned that the following were facilitators and advantages to providing teletherapy: easier access to patients given the lockdown levels, a decreased risk of exposure to COVID-19 and reduced financial strain in terms of travel and hospital costs on the patient and family. The limited risk of exposure for SLPs was highlighted as a key advantage to the introduction of teletherapy, as sessions were being held remotely. In addition, the use of teletherapy reduced the need for PPE, thus saving on costs for the patient and family as well as the SLP:

‘Less risk to patient in terms of potentially contracting infection such as [*COVID-19*].’ (Participant 4)

Improved family involvement in the rehabilitation process was noted, and research further highlights that teletherapy allows for fewer constraints on time, as sessions can be planned around the household schedule, and limits the time taken to travel, thus enabling more members of the family to participate. Furthermore, it allows the therapist to see the family in their living space, which is their most natural environment (Burgoyne & Cohn, 2020). This was seen by participants as family members were forced to take more responsibility and participate as facilitators in the therapeutic environment:

‘Very positive! All have participated, asked questions, reviewed sessionals and did extra. I’m so happy with how the caregivers are helping.’ (Participant 1)

‘Very involved and assist greatly. It has made the integration of caregivers into therapy much easier. It has been very very useful and beneficial.’ (Participant 3)

## Barriers and disadvantages of providing speech, language, swallowing intervention via teletherapy

In the context of South Africa, technology problems such as unreliable electricity supply and low bandwidth were identified as barriers to the successful implementation of telemedicine (Cilliers & Flowerday, 2014). This is seen clearly in the experiences of the SLPs, where load-shedding and connection difficulties were seen as barriers to service delivery. Research suggests that high Internet speed affects the quality of the consultation and can positively influence patients’ acceptance of and satisfaction with teletherapy (Almathami, Win, & Vlahu-Gjorgievska, 2020).

It is known that contextual relevance is important in health care, especially when it comes to establishing infrastructure and rolling out technology to promote health services. In a context like South Africa, where social determinants of health have contributed both to the inequitable distribution of health resources and access and to failure in redressing the injustices of the past, careful consideration must be taken to ensure that these service delivery models are both feasible and sustainable (Govender & Mars, 2018). Based on the participants’ responses below on the disadvantages, it is seen that teletherapy might not always be feasible in the context of South Africa where there are financial constraints, which can be further exacerbated by the need for data, technological devices and abilities for teletherapy:

‘Cost of data, patients are already struggling to pay for bare necessities. Lack of access to technology.’ (Participant 7)

Furthermore, the SLPs felt like therapy was less personal and like there was a lack of rapport-building, which is important to consider as an SLP as clinician–client relationships may influence SLP treatment success (Ebert, 2017). These were considered disadvantages for the SLPs, as seen below:

‘There is a restriction in the naturalness of the interaction and somehow you do not connect as easily with the individual.’ (Participant 4)

Research shows that it is difficult for both clients and therapists to enter a therapy mindset when using teletherapy at home, as there is a sequence that typically precedes and follows face-to-face therapy that does not take place with teletherapy for clients (Burgoyne & Cohn, 2020). This allows for distractions in the environment that were experienced by the SLPs in this study, as seen by Participant 4: ‘…noises and disturbances from people at home where my computer is’. Positive intervention for language-based disorders such as aphasia and cognitive-linguistic disorders was able to be conducted via teletherapy. Three factors were identified as important for their management: (1) family involvement, (2) good planning and (3) adaptation to resources, as seen below:

‘Require very good planning so that patient can have the right resources – use what they have at home.’ (Participant 2)

‘Much the same as face to face – just at times adaptations to therapy materials need to be made.’ (Participant 6)

The similarities between signs of aspiration as well as the symptomatology of COVID-19 highlighted the need for dysphagia service provision from admission until discharge and rehabilitation (SASLHA, 2020). Thus, making the provision of dysphagia services is vital; however, this seems to have been greatly affected because of the shift to teletherapy, as most of the SLPs were unable to do this via teletherapy. Participant 10 noted, ‘I am not doing any dysphagia treatment via teletherapy’.

## Access to teletherapy

In order to provide and receive teletherapy, one needs to have access to both devices and data. When looking at the client’s ability to access teletherapy services and the SLP’s ability to provide teletherapy services, one can expect major discrepancies given the South African context. A stark division was noted between the public and private sectors, which places emphasis on the country’s social divide. However, this must be interpreted with caution, as there were only three SLP participants who worked in the public sector.

Literature suggests that challenges experienced in the public sector are well documented and include poorly maintained facilities and reduced access to resources (Young, 2016). This directly links to the experience of the participants working in the public sector, as they have poor access to resources such as devices and data, as seen below:

‘I have to use my own money to acquire data and are not reimbursed by the government.’ (Participant 11)

‘Very limited resources available in the government sector. So, I do not have access to devices.’ (Participant 12)

Private health care is very different to public health care in South Africa in that it is at an advantage in terms of better facilities and availability of adequate resources (Young, 2016). This again directly links to the experiences of the SLPs working in the private sector, as they have better access to resources such as access to devices and data, as seen below:

‘Access to data is not a concern for me.’ (Participant 4)

‘I have a work laptop and can access appropriate platforms for telehealth. At work, there is great internet connection.’ (Participant 8)

Similar trends were noted with regard to the patients being seen for teletherapy. Participants reported that patients had poor access to devices across both private and public sectors:

‘I’ve done therapy over a WhatsApp voice call and even through a neighbour’s phone.’ (Participant 1)

‘The community I service are very poor and cannot afford to acquire devices or data to do online therapy.’ (Participant 12)

In terms of access to data, it was found that clients from the public sector had limited access whilst the majority of those in the private sector had access. This is backed up by the responses where a participant working in the private sector stated that ‘[c]lients have Wi-Fi which help in terms of having good connection for the duration of the session’ (Participant 8), in contrast to a participant working in the public sector who stated that ‘[l]ow income equals no money to buy data. It is a challenge’ (Participant 12).

Factors such as socio-economic status, culture, education and income, as well as access to information and communication technologies, their associated infrastructure and a stable electricity supply need to be taken into account. The technology gap between the public and private sectors is a main factor that impacted the service provision of many SLPs. In addition, issues such as unreliable electricity supply as well as cost of data and access to Wi-Fi are identified as barriers to the successful implementation of teletherapy (Cilliers & Flowerday, 2014). Research suggests that high Internet speed affects the quality of the consultation and can positively influence patients’ acceptance of and satisfaction with teletherapy (Almathami et al., 2020).

Geographic location is known to cause inconsistencies in accessing therapeutic services, as it has implications in terms of time and money on the patient. The implementation of teletherapy has shown to eliminate the need to travel long distances, and treatment can be instantaneous. Additionally, families responded positively when asked about their willingness to participate in future telehealth visits.

The treatment of apraxia and dysarthria were identified to be dependent on severity of the disorder, particularly in the private sector:

‘The approach mainly stays the same depending on the severity of the apraxia or dysarthria. The patients I see do well with visual and phonemic cueing. Surprisingly, this is easier over telehealth.’ (Participant 8)

The same treatment was more challenging in the public sector because of teletherapy being done telephonically rather than via video conferencing platforms. Participant 12 stated, ‘This is challenging as the patient cannot see your mouth over the telephone when trying to model the strategies, techniques, etc.’ This links back to the stark contrast seen in the public and private sectors in South Africa in terms of access to resources (Young, 2016).

From the above responses, it can be seen that the shift to teletherapy has had to become the new normal for practicing SLPs in South Africa because of COVID-19; however, it is met with great challenges that need to be addressed in order to ensure adequate access and equity of service provision.

## Conclusion

The study provided general insights into the transition from face-to-face therapy to teletherapy and the experiences of SLPs in this regard for stroke care. It further highlighted how practices in speech–language pathology have changed because of the COVID-19 pandemic. Furthermore, results from this study can inform practices with regard to management of stroke-related speech, language and swallowing difficulties using teletherapy in South Africa and thus develop guidelines on this new practice.

Despite the small sample size, results of this study provide important insights into SLPs’ experiences with regard to the use of teletherapy for rehabilitation post stroke. Speech–language pathologists who engaged in teletherapy reported on a variety of advantages and facilitators as well as disadvantages and barriers to service provision. Speech–language pathologists in the private sector had different experiences in terms of access to data and devices compared to SLPs in the public sector, and the overall SLP experiences differed. Essentially, it is seen that teletherapy, whilst challenging at times, can provide surprising benefits such as improved family involvement. We do not yet understand how the COVID-19 pandemic will impact our lives in the long term; however, given the importance of providing therapy services to patients after a stroke, it is important to establish best practices to ensure that clients’ needs are being met in both the private and public sectors.

## Appendix 1: Questionnaire

1. What province are you currently practicing in?
  - Gauteng
  - KwaZulu-Natal
  - Western Cape
  - Limpopo
  - Eastern Cape
  - Mpumalanga
  - North West
  - Free State
  - Northern Cape
2. How many years of experience do you have in the field of speech–language pathology?
  - 1–5 years
  - 5–10 years
  - More than 10 years
3. Which sector are you currently working in?
  - Public
  - Private
4. Approximately how many stroke patients do you see in a 6-month period?
5. Are you currently making use of or have you previously made use of teletherapy in providing services for speech, language and swallowing difficulties for patients after a stroke?
6. What do you think are some of the advantages of teletherapy?
7. What do you think are some of the disadvantages of teletherapy?
8. What are your experiences using teletherapy in terms of patient participation?
9. What are your experiences with patient outcomes using teletherapy in terms of teletherapy vs. face-to-face?
10. What are your experiences with your clients in terms of access to devices and being able to use these devices?
11. What are your personal experiences in terms of access to devices and being able to use these devices?
12. What are your experiences with your clients in terms of access to data?
13. What are your personal experiences in terms of access to data?
14. What are your experiences with caregivers and their involvement in the teletherapy process?
15. What are your experiences with managing dysphagia, and how has this changed now that you have changed to teletherapy?
16. What are your experiences with managing aphasia, and how has this changed now that you have changed to teletherapy?
17. What are your experiences with managing motor speech disorders (dysarthria and apraxia), and how has this changed now that you have changed to teletherapy?
18. What are your experiences with managing a cognitive-linguistic deficit, and how has this changed now that you have changed to teletherapy?
19. What are your experiences in terms of the multidisciplinary team and being able to include aspects of co-treatment, now that you are making use of teletherapy?
20. What are your experiences with counselling the patient and their family post stroke using teletherapy?
21. What were your main successes with teletherapy?
22. What were your main challenges with teletherapy?
